# Emotions and emotion regulation in survivors of childhood sexual abuse: the importance of “disgust” in traumatic stress and psychopathology

**DOI:** 10.3402/ejpt.v5.23306

**Published:** 2014-06-03

**Authors:** Eimear Coyle, Thanos Karatzias, Andy Summers, Mick Power

**Affiliations:** 1 Clinical and Health Psychology, University of Edinburgh, Edinburgh, UK; 2 Clinical Psychology Department, NHS Fife, Fife, UK; 3 Faculty of Health, Life and Social Sciences, Edinburgh Napier University, Edinburgh, UK; 4 Rivers Centre, Royal Edinburgh Hospital, Edinburgh, UK

**Keywords:** Childhood sexual abuse, emotion, PTSD

## Abstract

**Background:**

Childhood sexual abuse (CSA) has the potential to compromise socio-emotional development of the survivor resulting in increased vulnerability to difficulties regulating emotions. In turn, emotion regulation is thought to play a key part in a number of psychological disorders which CSA survivors are at increased risk of developing. A better understanding of the basic emotions experienced in this population and emotion regulation strategies will inform current treatment.

**Objective:**

This paper examines the relationships between type of emotions experienced, emotion regulation strategies, and psychological trauma symptoms in a sample of survivors of CSA.

**Method:**

A consecutive case series of CSA survivors (*n*=109) completed the Basic Emotions Scale (BES)—Weekly, General, and Coping versions; the Regulation of Emotions Questionnaire; the Post-traumatic Stress Checklist—Civilian Version (PCL-C); and the Clinical Outcomes in Routine Evaluation Outcome Measure.

**Results:**

Significantly higher levels of disgust than other levels of emotions were reported on the weekly version of the BES. In addition, significantly higher levels of disgust and lower levels of happiness were reported on the BES—General subscale. Regression analyses revealed that sadness, fear, disgust, and external dysfunctional coping strategies predicted global post-traumatic stress disorder and re-experiencing symptomatology measured by the PCL-C. Global distress, as measured by CORE, was predicted by the emotions of sadness, disgust, and low happiness, as well as dysfunctional regulatory strategies. In addition, preliminary exploratory factor analyses supported the structure of all three versions of the BES, with disgust explaining the largest percentage of variance, followed by happiness.

**Conclusions:**

The findings highlight the utility of profiling basic emotions in understanding the strong associations between emotional phenomena, particularly the emotion of disgust and psychopathology in CSA survivors.

Childhood sexual abuse (CSA) involves an interpersonal betrayal of trust, often in primary relationships, during critical developmental periods. This has the potential to compromise the socio-emotional development of the survivor resulting in increased vulnerability to difficulties in regulating emotions, attachment, and one's sense of self (Courtois & Ford, 2009). CSA has been recognised as a substantial risk factor for psychopathology, even when other childhood adversities have been controlled for (Molnar, Buka, & Kessler, 2001). Indeed, a range of psychological difficulties and disorders have been associated with CSA including anxiety disorders, post-traumatic stress disorder (PTSD), depressive disorders, and personality disorders (Spataro, Mullen, Burgess, Wells, & Moss, 2004).

In the past 15 years, there has been increased interest in the experience of everyday emotions and their relationship with psychopathology (Carolan & Power, 2011; Fox & Froom, 2009; Power, 2006; Power & Dalgleish, 1997; Power & Tarsia, 2007). This follows a long history of debate regarding the nature of emotion. Functionalist accounts of emotion suggest that “basic emotions” underlie emotional experience and related behaviour (Ekman, 1992; Oatley & Johnson-Laird, 1987). A basic group of emotions has been proposed which includes anger, disgust, anxiety, happiness, and sadness (Oatley & Johnson-Laird, 1987; Power & Dalgleish, 1997). A recent study examined how basic emotion profiles can differentiate clinical groups from non-clinical groups (Finucane, Dima, Ferreira, & Halvorsen, 2012). The PTSD group in this study was discriminated from healthy, chronic pain, and depressed groups by their experience of disgust and more frequent reporting of negative emotions (Finucane et al., 2012).

Power (2006) found confirmatory evidence for the proposed set of five emotions in a study involving a student sample who completed the Basic Emotions Scale (BES; Power, 2006). The study also provided support for a model of emotions known as the Schematic Propositional Analogical Associative Representation Systems (SPAARS) model (Power & Dalgleish, 1997, 2008). In brief, the SPAARS model proposes that each basic emotion is linked to a distinct appraisal of an event, and the emotion triggered by the appraisal signals the individual to act. SPAARS (Power & Dalgleish, 1997) is a multilevel model. Emotion-related stimuli are first processed by the Analogical system. The output is then processed, in parallel, by either the Associative (automatic emotion processing), Schematic (processing requires effortful appraisal), or the Propositional system (which indirectly operates via connection with the schematic and associative levels).

Emotional profiles can be shaped by feedback loops which develop either between or within basic emotion modules. Furthermore, the SPAARS model proposes that emotional disorder can be understood to be the result of the coupling of basic emotions, or processing levels, within the same emotion (e.g., associative and schematic levels). For example, the coupling of sadness and disgust is thought to underlie depression (Power & Dalgleish, 2008). Carolan and Power (2011) tested the predictions of the SPAARS model in a population of individuals diagnosed with bipolar disorder; depression would be characterised by sadness and disgust, and mania would be characterised by the coupling of happiness and anger. The findings supported these predictions, along with elevated levels of disgust in the bipolar population outwith episodes of depression or mania.

Fox and Harrison (2008) examined the coupling of emotions in eating psychopathology. They concluded that disgust and anger are likely to be coupled in bulimic pathology. The finding that psychopathology can be predicted by basic emotions has been supported by subsequent studies (Carolan & Power, 2011; Overton, Markland, Taggert, Bagshaw, & Simpson, 2008). Of interest to this study are the emotions which may underlie psychopathology in survivors of CSA. PTSD has been associated with CSA; however, several theorists have proposed that Diagnostic and Statistical Manual of Mental Disorders, Fourth Edition (DSM-IV; American Psychiatric Association, 2000) and Diagnostic and Statistical Manual of Mental Disorders, Fifth Edition (DSM-5; American Psychiatric Association, 2013) criteria for PTSD do not account for the full complexity of post-traumatic symptomatology observed in survivors of CSA. Disorders of extreme stress not otherwise specified (DESNOS), complex post-traumatic stress disorder (CPTSD), or *complex trauma* have been suggested as separate nosological entities from PTSD (Courtois & Ford, 2009; Herman, 1992; Knefel & Lueger-Schuster, 2013, Maercker et al., 2013). The SPAARS model suggests that emotion coupling of fear and disgust underlies some forms of PTSD (Power & Dalgleish, 2008; Power & Fyvie, 2013). The current study seeks to explore whether there is a more complex or different emotional picture underlying PTSD, when it is associated with CSA, in keeping with the idea that a more complex range of post-traumatic symptoms is experienced.

The SPAARS model proposes that the basis of PTSD is the cognitive system's inability to resolve inconsistencies between trauma-related information and the content of pre-existing mental representations (Dalgleish & Power, 2004). This discrepancy is thought to result in the PTSD symptom pattern of re-experiencing and avoidance of trauma-related information due to threat-based appraisals, with fear being the dominant emotion. Furthermore, Dalgleish and Power (2004) argue for a theoretical model of PTSD consisting of emotion-specific (e.g., disgust and fear) and emotion-non-specific components (e.g., avoidance and re-experiencing). They suggest that there might be a family of PTSD-like psychological reactions to extreme events which share emotion-non-specific components (re-experiencing and avoidance) but differ in terms of their emotion-specific component (emotions other than fear), for example, complex grief. It is, therefore, possible that the interpersonal nature of CSA-related trauma might result in different appraisals and different emotion-specific components, whilst retaining the non-emotion-specific components (e.g., the symptoms of re-experiencing and avoidance).

## Objective

The relationship between emotional phenomena and psychopathology in CSA survivors has not been studied previously. The purpose of this study is to investigate the emotions reported by individuals who have experienced CSA and their associations with traumatic and general distress symptoms. Considering the above, the aims of this study are to examine the following hypotheses:

**Hypothesis 1:** The CSA sample in this study will experience high levels of negative emotions; in particular, disgust is likely to be frequently experienced (Finucane et al., 2012; Power & Dalgleish, 2008).

**Hypothesis 2:** Basic emotions and emotion regulation strategies will predict post-traumatic symptomatology and general psychopathology.

Difficulties regulating emotions are a primary criterion in the proposed diagnostic criteria for CPTSD by the International Classification of Diseases (11th revision) (ICD-11) working group for mental disorders specifically associated with stress (Maercker et al., 2013). Emotion regulation difficulties have been associated with prolonged exposure to trauma (Cloitre et al., 2009, Herman, 1997). The contribution of emotion regulation difficulties to a model of emotion which predicts post-traumatic and general psychopathology will therefore be investigated.

## Method

### Design

The study used a quantitative cross-sectional consecutive case series design. Anonymous data were routinely collected from 109 individuals, at the point of referral to a psychotherapy service specialising in the treatment of adult survivors of CSA. Individuals were sent a pack of measures with a covering letter. The anonymous return of these measures was deemed a satisfactory indication of consent. Participation was voluntary; the return of questionnaires was not monitored and had no effect on treatment. Eighty-five out of 194 individuals did not return the forms. Ethical approval was provided by the appropriate Ethics Committee.

### Measures

#### Basic Emotions Scale

The BES (Power, 2006) is a three-part questionnaire which assesses basic state emotions (experienced over the past week) and trait emotions (experienced “in general”) and one's ability to cope with each of the 21 emotion terms listed. Each part of the BES uses a 7-point Likert scale for responses. The 21 emotion terms can be reduced to five subscales, which correspond to the five basic emotions (anger, sadness, disgust, fear, and happiness) as described by Oatley and Johnson-Laird (1987) and Power and Dalgleish (1997). Good internal reliability and discriminant group validity have been indicated in a sample of outpatients with anxiety and depression (Power & Tarsia, 2007). The validity of this measure was explored further in preliminary analysis in this study (see data analysis and results sections).

#### Regulation of Emotions Questionnaire

The Regulation of Emotions Questionnaire (REQ; Phillips & Power, 2007) is a 21-item self-report measure originally designed to assess the frequency with which adolescents employ functional and dysfunctional strategies, using internal and external resources to manage their emotions. Participants are required to indicate, using a 5-point Likert scale, how often they use a list of emotion regulation strategies. The items map onto four subscales—external functional (e.g., “I talk to someone about how I feel”), external dysfunctional (e.g., “I bully other people…”), internal functional (e.g., “I review (re-think) my thoughts or beliefs”), and internal dysfunctional (e.g., “I harm or punish myself in some way”). The validity of this measure was supported in a study of adolescents.

#### PTSD Checklist—Civilian Version

The PTSD Checklist—Civilian Version (PCL-C; Weathers, Litz, Herman, Huska, & Keane, 1993) consists of 17 items which correspond to the DSM-IV diagnostic criteria for post-traumatic stress. Participants identify how often they have been troubled by each symptom in the past month on a 5-point Likert scale. Psychometric data on the scale was originally established using the military version of the scale—reliability and validity have been demonstrated for clinical populations (Blanchard, Jones-Alexander, Buckley, & Forneris, 1996; Weathers et al., 1993).

#### Clinical Outcomes in Routine Evaluation

The Clinical Outcomes in Routine Evaluation Outcome Measure (CORE-OM; Evans et al., 2000) is a 34-item self-report measure of general psychological distress with scores on four dimensions—subjective well-being, problems or symptoms experienced, social functioning, and risk to self or others. Participants rate each of the 34 items on a 5-point scale (ranging from “not at all” to “most or all of the time”), indicating how they have felt over the week. Evans (2002) reports good internal consistency for all four dimensions across all domains, and good convergent validity with other standardised measures.

### Data analysis

A preliminary exploratory factor analysis of the BES was conducted to identify patterns of basic emotions experienced by CSA survivors and establish if the five basic emotions model proposed by Power and Dalgleish (2008) is supported in this population.

**Hypothesis 1:** One-way repeated measures Analysis of Variance (ANOVA) was conducted to assess for significant differences between the emotion subscales for the BES—Weekly and General scales.

**Hypothesis 2:** Regression analyses were conducted to determine if the PCL-C total scores and three subscales were predicted by *trait* basic emotions (sadness, fear, disgust, anger, and happiness on the BES—General scale) and REQ subscales, after adjusting for age and sex (see Table 3). A stepwise analysis was used to evaluate the predictive function of this set of variables on each of the dependent variables. For general distress, the outcome measures (dependent variables) were the CORE subscales. The independent variables were *state* basic emotions (sadness, fear, disgust, anger, and happiness) and the REQ subscales (internal dysfunctional, internal functional, external dysfunctional, and external functional). Sex and age were again included as background variables. A stepwise analysis was also used to evaluate the predictive function of this set of variables on each of the dependent variables.

## Results

### Preliminary analysis of the BES scale

#### The emotion structure—BES Weekly

Principal components extraction with varimax rotation of the 21 items measure resulted in the extraction of five components which had eigenvalues of 6.74, 2.82, 1.76, 1.24, and 1.13, respectively. All of these values exceeded the proposed minimum eigenvalue value of 1.0. Examination of the screen plot supported the retention of the five factors. Deeming all factor loadings that were over 0.50 as significant provided a five factor structure, which confirmed the factor structure of the BES. However, the “frustration” item did not load on any of the factors at this level but loaded on the “fear” factor (at 0.362). Reducing the acceptable factor loading significance level to 0.3 meant that “despair” cross loaded on the “fear” and “sadness” factors. Factor 1 (Disgust) accounted for 32.20% of the variance followed by Factor 2 (Happiness) which accounted for 13.45%; Factor 3 (Fear) also accounted for a high proportion of variance (8.41%).

#### The emotion structure—BES General

Five components were also extracted on this scale, with eigenvalues of 7.60, 3.09, 1.70, 1.44, and 1.04, accounting for 70.85% of the variance cumulatively. Consistent with the findings of the EFA of the BES Weekly, “disgust” was identified as the factor accounting for the most variance (36.19%). The “despair” and “frustration” items loaded on the “fear” subscale, which differs from the original scale construction of the BES.

#### The emotion structure—BES Coping

Five components were extracted with eigenvalues of 7.02, 3.05, 1.80, 1.41, and 1.08, accounting for 68.47% of the variance cumulatively. Deeming all factor loadings that were over 0.50 as significant gave a five factor structure, which confirmed the factor structure of the BES. However, this meant that the “frustration” and “irritation” items did not load on any factor. Reducing the factor loading significance level to 0.4 allowed both of these items to load onto Factors 1 and 4. However, at the level of 0.40 the “mournful” and “despair” items cross loaded onto Factors 3 (Fear) and 4 (Sadness).

#### Emotion and psychopathology

Tables 1 and 2 present data on the characteristics of the study sample including scores on emotion and symptom measures.

**Table 1:** Demographic and population characteristics

Factor	Level/units	Mean or *N* (sd or %) *n*=109
Age		35.5 (9.9)
Sex	Males	15 (13.8%)
	Females	85 (78%)
	Missing values	9 (8.3%)
Education	Basic education	42 (38.5%)
	Higher education	70 (45.8%)
	Missing values	17 (15.6%)
Employment	Full/part-time	40 (36.7%)
	Unemployed/retired/other	63 (57.8%)
	Missing values	6 (5.5%)
Marital status	Married/cohabiting	37 (35.0%)
	Divorced/single	65 (59.6%)
	Missing values	7 (6.4%)
Living arrangements	Alone	37 (33.9%)
	With others	66 (60.6%)
	Missing values	6 (5.5%)

**Table 2:** Means (sds) of emotion-related measures administered

Emotion measure	Mean (sd)	Symptom measure	Mean (sd)
BES—Weekly		PCL	
Anger	4.67 (1.2)		
Sad	4.63 (1.3)	Re-experience (Criterion B)	18.2 (5)
Disgust	4.64 (1.6)	Avoidance (Criterion C)	25.6 (5.9)
Fear	5.51 (1.1)	Hyperarousal (Criterion D)	18.1 (4.3)
Happy	3.25 (1.2)	Total	61.9 (13.4)
BES—General			
Anger	4.93 (1.2)		
Sad	4.78 (1.3)	CORE	
Disgust	4.77 (1.6)	Subjective well-being	3.0 (0.7)
Fear	5.70 (1.0)	Problems/symptoms	2.9 (0.8)
Happy	3.50 (1.3)	Functioning	2.4 (0.7)
BES—Coping		Risk	1.3 (0.9)
Anger	4.97 (1.2)	Global distress	2.5 (0.7)
Sad	5.12 (1.1)	Non-risk items	2.7 (0.7)
Disgust	5.31 (1.2)		
Fear	5.50 (1.0)		
Happy	3.33 (1.5)		
REQ			
External functional	2.30 (0.8)		
Internal functional	2.50 (0.7)		
External dysfunctional	2.10 (0.9)		
Internal dysfunctional	3.5 (0.7)		

BES=Basic Emotions Scale; REQ=Regulation of Emotions Questionnaire.

**Hypothesis 1:** One-way repeated measures ANOVA demonstrated a significant difference between the frequency of experience of the basic emotions reported (*F*=(2.70,291.60)=71.25, *p*<0.0001, η^2^=0.39). Post hoc tests using the Bonferroni correction demonstrated that significantly higher levels of disgust (23.22±8.31; *p*<0.01) were reported compared to all other emotions on the weekly version of the BES.

**Hypothesis 2:** For the BES—General scale, a significant difference was found for the five basic emotions subscales (*F*=(2.59,280.23)=65.88, *p*<0.001, η^2^=0.37). Post hoc tests using the Bonferroni correction demonstrated that the sample reported significantly higher levels of disgust (23.85±8.01) and lower levels of happiness (14.0±5.33) than any of the other three subscales (*p*<0.01).

#### Post-traumatic symptoms

Regression analyses produced three models for each of the subscales. The final model for each of the subscales is reported above. Age and sex were not significant predictors. Disgust was retained in the third model for PCL-C total scale but was not significant. Prior to the addition of the REQ subscales to the model, disgust was significant (*β*=0.35, *p*=0.039). The results suggest that sadness, fear, disgust, and external dysfunctional coping strategies predict global PTSD symptomatology. Re-experiencing is also predicted by the same three emotions (sadness, fear, and disgust). Hyperarousal is predicted by sadness, fear, and external dysfunctional strategies, and avoidance is predicted by sadness (Table 3).

**Table 3:** Predicting post-traumatic symptomatology from “trait” emotions and emotion regulatory strategies

	Post-traumatic Checklist—Civilian Version			
	
	PLC-total	Re-experiencing	Hyperarousal	Avoidance
Predictor	Beta	Beta	Beta	Beta
BES-G disgust	0.163	0.317***		
BES-G sad	0.329**	0.205*	0.335***	0.344***
BES-G fear	0.242*	0.287**	0.228**	–
REQ external dysfunctional	0.161*	–	0.337***	–
Adj. R^2^	0.445	0.445	0.415	26.9
F	20.81	27.44	24.43	19.19

BES-G=Basic Emotions Scale—General; REQ=Regulation of Emotions Questionnaire.

* *p*<0.05;

** *p*<0.01;

*** *p*<0.001.

### General psychopathology

The analyses produced five models, the final model retained “disgust,” but not as a significant predictor on the global distress or subjective well-being subscales. Disgust was a significant predictor for global distress (*β*=0.024, *p*=0.002) and subjective well-being (*β*=0.024, *p*=0.01) prior to the REQ subscales being entered into the regression model. The results suggest that global distress (with and without risk) in this population is predicted by the emotions of sadness and disgust, and negatively predicted by happiness and dysfunctional regulatory strategies. Problems on the CORE are predicted by sadness, fear, and low happiness. Disgust is the only emotion in the model predicting risk. Disgust was a significant predictor (*p*<0.001) for the risk subscale, until the regulatory strategies were entered into the model (Table 4).

**Table 4:** Significant predictors of general psychopathology on the CORE from “state” emotions and emotion regulatory strategies—final model

	CORE					
	
	Global distress	Global distress-risk	Problems	Subjective well-being	Functioning	Risk
Predictor	Beta	Beta	Beta	Beta	Beta	Beta
BES-W disgust	0.137	0.201*		0.174	0.203**	0.168
BES-W happy	−.261***	−.248***	−.223**	−.329***	−.179*	
BES-W sad	0.293***	0.273**	0.239*	0.211*	0.301***	
BES-W fear			0.184*			
REQ external functional					−.275***	
REQ external dysfunctional	.195**	−.171*			0.217**	0.288**
Adj. R^2^	0.581	0.528	0.488	0.460	0535	0.415
F	28.50	23.95	19.90	22.10	23.79	18.58

BES-W=Basic Emotions Scale Weekly; REQ=Regulation of Emotions Questionnaire; CORE=Clinical Outcomes in Routine Evaluation.

* *p*<0.05;

** *p*<0.01;

*** *p*<0.001.

## Discussion

The results of this study provide support for the significance of emotional experience in the symptomatology of survivors of CSA. Consistent with previous research (Finucane et al., 2012), high levels of self-reported negative emotions were observed as well as high levels of PTSD in our sample. The exploratory factor analysis was consistent with the SPAARS model profile, comprising of five basic emotions. The results indicate that the BES provides a clinically relevant factor structure in CSA survivors. There was a consistent pattern across all three scales with the largest proportion of variance accounted for by “disgust,” followed by “happiness” and “fear.” The results lend additional support for previous findings of the utility of the BES in a student sample and clinical population of depressed and anxious individuals (Power, 2006; Power & Tarsia, 2007).

Disgust explained the highest percentage of variance for all three versions of the BES, followed by happiness. This is consistent with other research in the field where elevated levels of disgust have been observed in individuals with a diagnosis of PTSD (Finucane et al., 2012). Furthermore, women who have a history of CSA and resultant PTSD report significantly more disgust during recall of the incident, than those without PTSD (Shin et al., 1999). Until relatively recently, disgust has been an emotion overlooked in the literature, and shame has often been a focus when considering psychopathologies such as depression and eating disorders (Phillips, Senior, Fahy, & David, 1998; Power & Dalgleish, 1999). These findings provide additional evidence of the importance of disgust in the treatment of mental health difficulties in trauma populations.

### Emotion and PTSD

Sadness, fear, disgust, and external dysfunctional coping strategies predicted global PTSD symptomatology in our study. Hyperarousal was predicted by sadness, fear, and external dysfunctional strategies, and avoidance was predicted by sadness. These findings provide support for the coupling of disgust and fear in post-traumatic symptomatology. However, the finding that sadness and external dysfunctional regulatory strategies also predict a large proportion of variance is interesting. There are two possible explanations for this finding. First, it is possible that sadness was a predictor because participants had high levels of co-morbid depression. Depression was not assessed in this sample. Previous researchers have highlighted the difficulties associated with differentiating between depression and PTSD in survivors of CSA (Wolfe & Kimerling, 1997). It is often considered difficult to establish whether depressive symptoms constitute a separate disorder, or a response to post-traumatic symptomatology (Wolfe & Kimerling, 1997).

Increased depressive symptomatology has been associated with CSA (Briere & Runtz, 1988; Browne & Finkelhor, 1986; Elliott & Briere, 1992). Briere (1996) attributes the increased levels of depression to distortions in cognitions relating to self, others, the world, and the future, which occur as a child in an attempt to make sense of the trauma. These distortions are thought to be maladaptive in adulthood when they become internalised, resulting in depression. It is possible that the finding that sadness predicts PTSD symptoms, alongside fear, disgust, and external dysfunctional regulatory strategies, may explain co-morbid depression in PTSD.

Second, the finding of sadness, as well as disgust and fear, as a predictor may point to a differential emotional component in CSA-related PTSD. Sadness is an emotion traditionally associated with the appraisal of loss (Power & Dalgleish, 1997). One might hypothesise that there are many different kinds of loss associated with interpersonal traumas such as CSA; for example, a loss of trust, childhood, and a sense of self (Sofka, 1999). It would be plausible, therefore, to conceptualise CSA-related PTSD, as a disorder which has a non-emotion-specific component consistent with the traditional model of PTSD (re-experiencing and avoidance), and a different emotion-specific component, that is, sadness coupled with fear and disgust. Research highlights the salience of sadness in the psychological sequelae of CSA. Conway, Mendelson, Giannopoulos, Csank, and Holm (2004) report increased levels of rumination on sadness in a population of CSA survivors, and an association between severity of abuse and levels of rumination. In addition, Brewin, Hunter, Carroll, and Tata (1996) found an association between intrusive memories related to CSA and depression. Dalgleish and Power (2004) label a “sadness emotion component” as a “traumatic loss reaction.”

The SPAARS model proposes that there are two routes to emotion; associative and schematic routes, which can operate in parallel (Power & Dalgleish, 2008). Interpreting findings from the current study within the SPAARS model might suggest that CSA-related PTSD may result in disgust and fear being processed through an associative route (the individual has learned that interpersonal experiences are threatening, or to be feared), whilst sadness may be generated via the schematic route (where childhood and interpersonal experiences are appraised in terms of traumatic loss and there are increased levels of rumination Conway et al., 2004). This hypothesis might have important implications for therapeutic approaches because, in theory, one could therefore address fear at the associative level using behavioural approaches, and use cognitive therapy to address appraisals of loss. It is proposed that therapy addressing emotions at associative and schematic levels for PTSD and other psychopathology could be examined in a study which dismantles the components of therapy in this population. Disgust has been shown to be a more difficult emotion to address using exposure, than fear (Olatunji, Lohr, Sawchuk, & Tolin, 2007; Smits, Telch, & Randall, 2002). A dismantling study which examined the effect of targeting disgust at the associative level using behavioural strategies versus cognitive therapy aimed at the schematic level would be a clinically relevant approach to improving the evidence base.

With regard to clinical relevance and emotion associated with PTSD, it is also interesting to note that the recently published DSM-5 includes PTSD in the “Trauma- and Stressor-related Disorders” chapter rather than “Anxiety Disorders” and includes reference to negative cognitions.

### Emotion and general psychopathology

In terms of general psychopathology, global distress was predicted by the emotions of sadness and disgust, and negatively predicted by happiness and external dysfunctional regulatory strategies. This finding has significant clinical implications; it may be that by attempting to reduce external dysfunctional regulatory strategies in therapy, distress might increase as these strategies (although considered dysfunctional) serve an important purpose in managing affect. Problems on the CORE were predicted by sadness, fear, and (low) happiness. Interestingly, disgust was the only emotion included in the model predicting risk. Power and Dalgleish (2008) suggest that disgust may play an important role in suicide and parasuicide. Similar to the findings for the post-traumatic symptoms, sadness, fear, and disgust are salient emotions associated with distress and functioning in this sample of CSA survivors.

### Emotion regulation strategies and psychopathology

There is much evidence to suggest that individuals who survive CSA experience difficulty regulating their emotions (e.g., Cloitre, Miranda, Stovell-McClough, Chase, & Han, 2005). The findings in this study that dysfunctional regulatory strategies contribute to psychopathology including PTSD, global distress, risk, and functioning is consistent with previous research (Cloitre et al., 2005) and highlights the value of addressing emotion regulation in therapy.

### Strengths and limitations of the study

There are a number of limitations associated with this study. First, confirmatory factor analyses are required to allow solid conclusions to be drawn about the emotional profile of CSA survivors. Second, although a measure of general distress has been used, it would have been useful to have included a measure of depression symptoms. Strengths of this study include that the findings highlight the importance of the emotion of disgust in understanding psychopathology associated with CSA, and that a model which accounts for the experience of everyday emotion, as well as emotional disorders, has been used as the basis of the hypotheses.

## Conclusions

Psychological therapies for survivors of CSA may need to focus on incorporating emotion regulation skills, prior to or alongside addressing emotional change on a schematic or associative level, in order to tackle psychopathology. Interestingly, “Third Wave” cognitive therapies, such as Dialectical Behavioural therapy, which have demonstrated effectiveness in treating Borderline Personality disorder (BPD) and success in treating CSA survivors with co-morbid BPD or depression, already take this approach, emphasising the development of emotion regulation skills (Linehan, 1993; Linehan et al., 2006; Steil, Dyer, Priebe, Kleindienst, & Bohus, 2011). Furthermore, acknowledgement of the specific emotions implicated in psychopathology is important in formulation and treatment.
